# *Leishmania* infection alters macrophage and dendritic cell migration in a three-dimensional environment

**DOI:** 10.3389/fcell.2023.1206049

**Published:** 2023-07-28

**Authors:** Yasmin Luz, Amanda Rebouças, Carla Polyana O. S. Bernardes, Erik A. Rossi, Taíse S. Machado, Bruno S. F. Souza, Claudia Ida Brodskyn, Patricia S. T. Veras, Washington L. C. dos Santos, Juliana P. B. de Menezes

**Affiliations:** ^1^ Laboratory of Host—Parasite Interaction and Epidemiology, Gonçalo Moniz Institute, Salvador, Brazil; ^2^ Center for Biotechnology and Cell Therapy, São Rafael Hospital, Salvador, Brazil; ^3^ D’Or Institute for Research and Education, Salvador, Brazil; ^4^ Laboratory of Tissue Engineering and Immunopharmacology, Gonçalo Moniz Institute, Salvador, Brazil; ^5^ Laboratory of Structural and Molecular Pathology, Gonçalo Moniz Institute, Salvador, Brazil

**Keywords:** macrophages, dendritic cells, 3D migration, *Leishmania*, dissemination

## Abstract

**Background:** Leishmaniasis results in a wide spectrum of clinical manifestations, ranging from skin lesions at the site of infection to disseminated lesions in internal organs, such as the spleen and liver. While the ability of *Leishmania*-infected host cells to migrate may be important to lesion distribution and parasite dissemination, the underlying mechanisms and the accompanying role of host cells remain poorly understood. Previously published work has shown that *Leishmania* infection inhibits macrophage migration in a 2-dimensional (2D) environment by altering actin dynamics and impairing the expression of proteins involved in plasma membrane-extracellular matrix interactions. Although it was shown that *L. infantum* induces the 2D migration of dendritic cells, *in vivo* cell migration primarily occurs in 3-dimensional (3D) environments. The present study aimed to investigate the migration of macrophages and dendritic cells infected by *Leishmania* using a 3-dimensional environment, as well as shed light on the mechanisms involved in this process.

**Methods:** Following the infection of murine bone marrow-derived macrophages (BMDM), human macrophages and human dendritic cells by *L. amazonensis*, *L. braziliensis*, or *L. infantum*, cellular migration, the formation of adhesion complexes and actin polymerization were evaluated.

**Results:** Our results indicate that *Leishmania* infection inhibited 3D migration in both BMDM and human macrophages. Reduced expression of proteins involved in adhesion complex formation and alterations in actin dynamics were also observed in *Leishmania*-infected macrophages. By contrast, increased human dendritic cell migration in a 3D environment was found to be associated with enhanced adhesion complex formation and increased actin dynamics.

**Conclusion:** Taken together, our results show that *Leishmania* infection inhibits macrophage 3D migration, while enhancing dendritic 3D migration by altering actin dynamics and the expression of proteins involved in plasma membrane extracellular matrix interactions, suggesting a potential association between dendritic cells and disease visceralization.

## 1 Introduction

Leishmaniasis is a neglected tropical disease endemic in 98 countries. Every year, it is estimated that between 700,000 and 1 million new cases occur, leading to approximately 30,000 deaths (Alvar et al., 2012). Cutaneous leishmaniasis (CL) in the New World, typically caused by *L. amazonensis* or *L. braziliensis*, results in ulcerative skin lesions. By contrast, visceral leishmaniasis (VL), which is caused by *L. infantum,* can affect the internal organs, such as the liver, spleen, and bone marrow, and may lead to more severe clinical manifestations, even death, if left untreated (Ashford, 2000; Desjeux, 2004; Kaye and Scott, 2011; Podinovskaia and Descoteaux, 2015).

Infection in the vertebrate host occurs through the inoculation of metacyclic promastigotes in the host’s skin by female sandflies during bloodfeeding (Veras and Bezerra de Menezes, 2016). Once inside the mammalian host, *Leishmania* parasites can survive and multiply inside acidic parasitophorous vacuoles within macrophages (Afrin et al., 2019). Subsequently, parasites may either persist at the local inoculation site or become disseminated to other tissues. The detection of *Leishmania*-infected mononuclear phagocytes in the bloodstream suggests that migration by infected macrophages may potentially contribute to the spread of disease (Liarte et al., 2001). Additionally, previous studies have shown that dendritic cells carrying *Leishmania* parasites can migrate, consequently transporting parasite antigens to lymph nodes (Ato et al., 2002). However, the mechanisms involved in the dissemination of *Leishmania* to different host tissues, as well as the homing and persistence of infected cells *in vivo,* remain poorly understood.

The cell migration carried out by a wide variety of cells plays an essential role in many physiological processes, such as leukocyte trafficking and immune response (Ridley and Hall, 1992; Ridley et al., 2003; Friedl and Wolf, 2010; Gardel et al., 2010). Integrated molecular events mediated primarily by actin filaments in the cytoplasm, in addition to the formation of adhesion complexes, are the main aspects involved in cell migration (Sheetz et al., 1999; Wiesner et al., 2014). Initially, a process mediated by integrins (Ridley et al., 2003), actin-binding proteins (talin, vinculin, and α-actinin) and adapter proteins (Paxillin, FAK, and Src) results in the formation of adhesion complexes (Turner, 2000; Zamir and Geiger, 2001), allowing the plasma membrane to anchor itself to the substrate. FAK protein functions as a focal adhesion regulatory factor during cell movement (Zaidel-Bar et al., 2004), leading to the phosphorylation of members of the Src family of tyrosine kinases, Paxillin, and p130Cas, thus creating binding sites for the formation of adhesion complexes (Toutant et al., 2002; Playford and Schaller, 2004; Mitra and Schlaepfer, 2006; López-Colomé et al., 2017). Additionally, proteins from the Rho family of small GTPases (Rac1, Cdc42, and RhoA) (Ridley and Hall, 1992; DeMali and Burridge, 2003; Huveneers and Danen, 2009; Huttenlocher and Horwitz, 2011; López-Colomé et al., 2017) are responsible for the regulation of actin dynamics within cells. Rac-1 controls the extension of lamellipodia in the anterior region of the cell, while the extension of filopodia is regulated by Cdc-42 (DeMali and Burridge, 2003; Huveneers and Danen, 2009; López-Colomé et al., 2017). RhoA controls retraction of the rear end of the cell, in addition to the formation of stress fibers throughout the cell structure (Ridley and Hall, 1992).

Previous studies have evidenced that *Leishmania* infection reduces macrophage adhesion by altering the function of molecules involved in adhesion (Carvalhal et al., 2004; Pinheiro et al., 2006). In addition, decreased macrophage migration has been observed following *Leishmania* infection in a two-dimensional environment (Blewett et al., 1971; Bray et al., 1983). A report published by de Menezes et al. (2017) also demonstrated a reduction in macrophage migration due to *L. amazonensis* infection in a two-dimensional environment, which was further associated with diminished adhesion complex formation and a greater frequency of polymerization and actin filament turnover (de Menezes et al., 2017). In contrast, a recent study published by our group identified increased human dendritic cell migration following infection with *L. infantum*, but not *L. braziliensis* and *L. amazonensis*, the latter two being localized forms of CL. Additionally, we found increased expression of proteins involved in adhesion complex formation and actin polymerization, as well as higher CCR7 expression in the human dendritic cells infected with all three isolates (Rebouças et al., 2021).

While most studies investigating cell migration are performed in a two-dimensional environment, the study of cellular infiltration into tissue requires the use of a three-dimensional environment. Within a 3D environment, leukocytes can migrate via amoeboid or mesenchymal modes (Friedl and Wolf, 2010; Huttenlocher and Horwitz, 2011; Wiesner et al., 2014). Each mode of migration is determined by the mechanical, structural, and biochemical properties of a given extracellular matrix. Amoeboid migration is dependent on the Rho/ROCK pathway, which is responsible for the regulation of actomyosin. In denser environments, the cell assumes a mode of mesenchymal migration dependent on the secretion of proteases and the formation of podosomes (Wiesner et al., 2014). In an attempt to better understand the mechanisms involved in the spread of *Leishmania* within the host, the present study aimed to evaluate the migration of *Leishmania* spp.-infected macrophages and dendritic cells through the use of a 3D environment.

## 2 Materials and methods

### 2.1 Ethics statement

The isogenic mice (*Mus musculus*) of the Balb/c strain used in the present study were provided by the animal care facility of the Gonçalo Moniz Institute (IGM-FIOCRUZ/BA), following approval by the Institutional Animal Experimentation Review Board (CEUA) under protocol number 014/2019.

Whole blood monocytes were isolated from healthy blood donors following approval by the Institutional Review Board of the Gonçalo Moniz Institute, Oswaldo Cruz Foundation (CEP/IGM-FIOCRUZ) (protocol No. 5.138.962).

### 2.2 *Leishmania* culturing

Promastigotes of *L. amazonensis* (MHOM/Br88/BA125), *L. braziliensis* (MHOM/BR/01/BA788), and *L. infantum* (MCAN/BR/89/BA262) were maintained for up to six successive passages in Schneider’s Insect Medium (Sigma- Aldrich, Saint Louis, MO, United States), or modified essential minimum medium hemoflagellate (MEM Medium), both supplemented with 50 μg/mL gentamicin (Gibco, Waltham, MA, United States) and 10%–20% (v/v) FBS (Gibco, Waltham, MA, United States). Promastigotes were grown in an incubator at 24°C and monitored daily by counting in a Neubauer chamber. Upon reaching stationary phase, promastigotes were then used in the experiments.

### 2.3 Mouse bone marrow‐derived macrophage cultures

Bone marrow‐derived macrophages (BMDM) obtained from Balb/c mice were cultured for 7 days in RPMI medium containing 10% FBS (Gibco, Waltham, MA, United States), 30% L929 cell supernatant containing macrophage colony-stimulating factor (M-CSF), 200 mM glutamine (Sigma-Aldrich, Saint Louis, MO, United States) and 10 μg/mL ciprofloxacin (Isofarma, Eusébio, CE, BR). Mature, adherent BMDM were detached using 0.05% EDTA/PBS (Gibco, Waltham, MA, United States). Cell viability was determined via trypan blue exclusion.

### 2.4 Human monocyte-derived macrophage and dendritic cell cultures

Human monocyte-derived macrophages were obtained from freshly isolated healthy peripheral blood monocytes using Ficoll–Histopaque density gradient separation (Sigma-Aldrich, Saint Louis, MO, United States). Peripheral blood mononuclear cells (PBMCs) were washed three times and then plated at 2 × 10^6^ in 500 mL of Roswell Park Memorial Institute (RPMI) supplemented with 25 mM N-[2-hydroxyethyl] piperazine-N′-[2-ethane sulfonic acid] (HEPES), 2 g/L sodium bicarbonate, 2 mM glutamine, 20 g/mL ciprofloxacin and 10% inactivated FBS (complete RPMI medium) for 7 days at 37°C under 5% CO_2_, on 24-well plates to allow monocytes to differentiate into macrophages. For human dendritic cells, peripheral blood monocytes were collected by positive selection using a magnetic cell sorter to isolate CD14^+^ subtypes. Positive cells were then plated in RPMI with granulocyte-macrophage colony-stimulating (GM-CSF) [50 ng/mL] and interleukin-4 (IL-4) [100 UI/mL] for 7 days to allow dendritic cell differentiation.

### 2.5 Preparation of 3D collagen matrix

Cells were embedded in a collagen type I matrix derived from rat tail collagen (Gibco), as described (Van Goethem et al., 2010). Cells (5 × 10^4^) were added to a solution containing collagen I [2 mg/mL], PBS 1X, and NaOH [2.6 mM] before polymerization at 37°C for 24 h.

To investigate the surface and shape of the 3D collagen matrix with embedded macrophages, scanning electron microscopy (SEM) was employed, following a previously published protocol (Cruz et al., 2021).

### 2.6 Bone marrow-derived macrophages, human macrophages and dendritic cell infection

BMDM and human macrophages were plated on 24-well plates for immunofluorescence (2 × 10^5^) and migration assays (5 × 10^4^) 24 h prior to experimentation. *L. braziliensis* (10:1) or *L. infantum* (20:1) promastigotes were added to both macrophage cultures and incubated for 24 h at 37°C, while cultures with added *L. amazonensis* (10:1) promastigotes were incubated for 6 h at 37°C. BMDM and human macrophages were then washed in PBS to remove any non-internalized parasites, and then re-incubated for 24 or 48 h for cell migration assays, or 24 h for immunofluorescence. For dendritic cells, *L. amazonensis* (10:1), *L. braziliensis* (10:1) and *L. infantum* (20:1) promastigotes were added to dendritic cell cultures and incubated for 4 h at 37°C. Cells were then washed in PBS to remove non-internalized parasites, then re-incubated in RPMI for 6, 12, 24, and 48 h (for BMDM and human macrophages) and for 6, 24, and 48 h (for dendritic cells) at 37°C.

### 2.7 Parasite burden

BMDM, human macrophages and dendritic cells were infected as described earlier. Subsequently, the cells were washed with PBS to remove non-internalized parasites and then either fixed (0 h) or re-incubated in RPMI at 37°C for 6, 12, 24, and 48 h (for BMDM and human macrophages) and for 6, 24, and 48 h (for dendritic cells). After the specified incubation period, coverslips were fixed using 4% paraformaldehyde, washed, and mounted using the ProLong Gold Antifade kit with DAPI (4′,6-diamidino-2-phenylindole) (Life Technologies). The number of intracellular parasites was determined by counting the total macrophages and the total intracellular parasites per microscopic field using a fluorescence microscope. At least 400 host cells, in triplicate, were analyzed for each time point.

### 2.8 Migration assay

BMDM, human macrophages, and dendritic cells were infected as previously described and then washed to remove non-internalized parasites. Following the infection period, BMDM (5 × 10^4^/well) were incorporated into a collagen I matrix (Gibco) and plated on 96-well plates for matrix polymerization. The matrices were then incubated for 24 or 48 h at 37°C. Time-lapse evaluations were then performed using an Operetta High-Content Analysis System (PerkinElmer, Waltham, MA, United States) at this timepoints, with image acquisition performed every 2 min for a period of 1 h to analyze cell migration rates. For human macrophages and human dendritic cells (5 × 10^4^), a chemotaxis assay was performed in Boyden chambers containing polycarbonate membranes (24-well, 5 µm pores, Corning^®^ Transwell^®^ polycarbonate membrane cell culture inserts). Each 3D collagen I matrix containing human macrophages or dendritic cells was incubated for 24 or 48 h. After this incubation period, the matrices were then placed within a Transwell insert, and allowed to migrate for an additional 4 h toward RPMI containing 100 ng/mL MCP1 (Monocyte chemoattractant protein- 1) for macrophages and 300 ng/mL CCL3 (C-C motif ligand 3) for dendritic cells. Next, the matrix was removed, and membranes were washed with PBS, fixed with 4% paraformaldehyde for 15 min, washed again with PBS and incubated with 10 mg/mL DAPI for nuclear staining. The upper side of the filter was scraped to remove any residual non-migrating cells. Cells in 10 random fields from each membrane were counted using a fluorescence microscope.

### 2.9 Immunofluorescence assay

Following migration, 3D collagen I matrices containing BMDM, human macrophages or human dendritic cells infected or not with *L. amazonensis, L. braziliensis,* or *L. infantum,* were fixed with 4% (v/v) paraformaldehyde for 15 min and washed five times in PBS. The matrices were then quenched for 20 min with 15 mM NH_4_Cl and washed five times with PBS, incubated in a pre-blocking solution (2% (v/v) bovine serum albumin (BSA) in 1% glycine) for 1 h, washed five times with PBS, permeabilized with 0.15% (v/v) Triton X-100/PBS (Sigma-Aldrich, Saint Louis, MO, United States) for 15 min, incubated in a blocking solution (1% BSA in PBS) for 45 min, and then incubated for 1 h with 1:500 rabbit anti-pFAK (0.5 μg/mL) (Invitrogen, catalog number RC222574), 1:100 rabbit anti-pPaxillin (0.1 μg/mL) (Invitrogen, catalog number QF221230), 1:200 rabbit anti-Rac1 (2.5 ng/mL) (BD Biosciences, catalog number 610650), 1:100 mouse anti-Cdc42 (1 ng/mL) (Invitrogen, catalog number PA1-092X), 1:100 rabbit anti-RhoA (1 μg/mL) (Invitrogen, catalog number 0SR00266W), mouse anti-Geosolin (1 μg/mL) (Sigma, catalog number G4896) or 1:1200 phalloidin (1 μg/mL) (Invitrogen, catalog number A12379). All antibodies were diluted in 1% (v/v) PBS +1% (v/v) BSA. Subsequently, anti-mouse Alexa fluor 594 (0.02 μg/μL) (Molecular Probes, catalog number A1011) or anti-rabbit Alexa fluor 488 (0.02 μg/μL) (Molecular Probes, catalog number A32732) was added and reincubated for 1 h. Cells were mounted using Prolong Gold antifade reagent with DAPI for nuclear staining (Invitrogen). Images were acquired using a Leica TCS SP8 confocal microscope with a 63x/1.4 objective. The Z-stack tool was employed with 0.3 μm intervals. Fiji software was used for digital fluorescence quantification. Maximal projection images were utilized, and the quantification considered the cell area. Several parameters, including Area, Min and Max gray value, integrated density, and mean gray value, were quantified for individual cells. To calculate the corrected total cellular fluorescence (CTCF), the following formula was applied: CTCF = integrated density - (selected cell area x mean fluorescence of background readings).

### 2.10 Statistical analysis

All experiments were repeated three times. After verifying data normality using D’Agostino, Kolmogorov-Smirnov and Shapiro-Wilk tests, the use of parametric or non-parametric tests for data analysis was determined. Parametric data were submitted to ANOVA testing (unpaired), while the Kruskal–Wallis test (unpaired) was used for non-parametric data. All analysis was performed using GraphPad Prism software. Results were considered significant when *p* < 0.05.

## 3 Results

### 3.1 Rates of macrophage infection over time, comparing *L. amazonensis*, *L. braziliensis* and *L. infantum*

**FIGURE 1:**
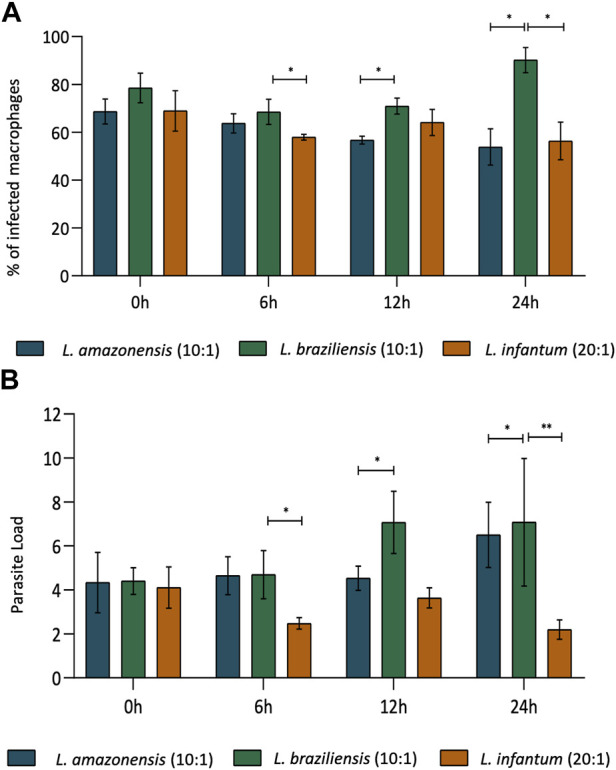
Rates of BMDM infection over time, *comparing L. amazonensis, L. braziliensis* and *L. infantum*. In BMDM infected by *L. amazonensis* (10:1), *L. braziliensis* (10:1), or *L. infantum* (20:1), 400 cells were randomly evaluated by fluorescence microscopy using DAPI. **(A)** Percentage of BMDM infected by *L. amazonensis, L. braziliensis*, or *L. infantum* at 0, 6, 12, and 24 h. **(B)** Mean number of *L. amazonensis, L. braziliensis,* or *L. infantum* amastigotes per BMDM at 0, 6, 12, and 24 h ***p,0.001; **p < 0,01; *p < 0,05 (ANOVA; Dunn’s multiple comparisons test). Representative results from three independent experiments.

The rates of BMDM infection were compared, considering *L. amazonensis* (10:1), *L. braziliensis* (10:1), and *L. infantum* (20:1), in an attempt to standardize the percentage of macrophages infection between species. Our results indicate that similar rates of infection (70%–80%) were achieved between macrophages infected by *L. amazonensis* (10:1), *L. braziliensis* (10:1), or *L. infantum* (20:1) at 0 h (Figure 1A). Accordingly, the number of *Leishmania* per macrophage was similar, with an average of 4 *Leishmania* per cell (Figure 1B). Similar results were found for human macrophages (Supplementary Figure S1).

### 3.2 *Leishmania* infection reduces macrophage migration in a 3D environment

**FIGURE 2:**
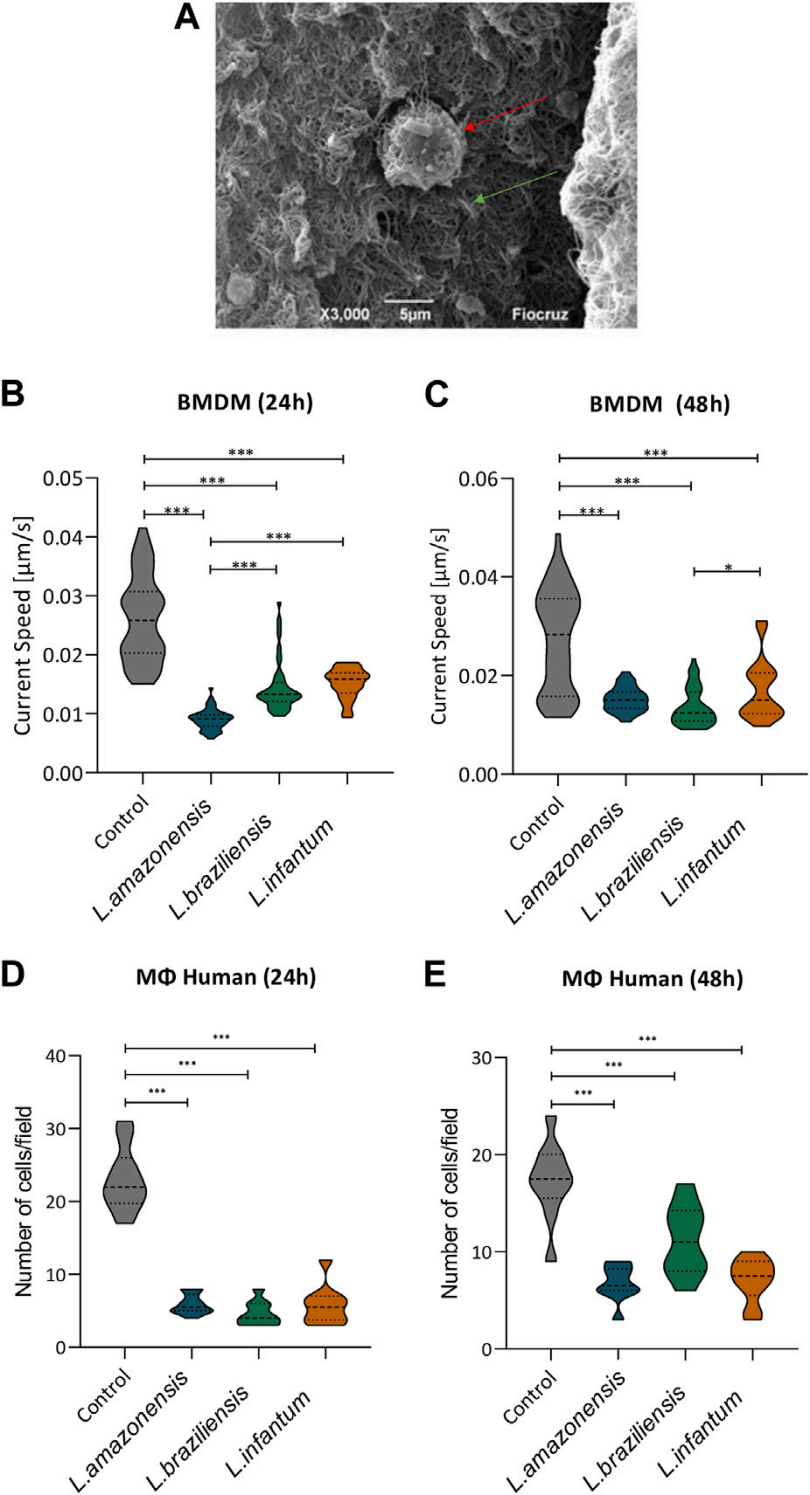
*Leishmania* reduces macrophage migration in a 3D environment. Macrophages infected by *L. amazonensis, L. braziliensis,* or *L. infantum* were incorporated into a collagen I matrix for 24 or 48 h. **(A)** Macrophage (red arrow) embedded within collagen I matrix (green arrow), Jeol 6390 Scanning Electron Microscope. Migration rates were calculated considering the trajectory of murine macrophages using time-lapse images obtained every 2 min over 1 h. **(B)** Analysis of murine macrophage migration rates after 24 h of infection. **(C)** Analysis of murine macrophage migration rates after 48 h of infection. **(D)** Numbers of human macrophages that migrated after 24 h in the presence of MCP-1. **(E)** Numbers of human macrophages that migrated after 48 h in the presence of MCP-1. ***, *p* < 0.001 (Kruskal Wallis). Representative results from three independent experiments.

To assess macrophage migration in a 3D environment, we incorporated BMDM into a collagen I matrix (Figure 2A) and allowed cells to migrate for 24 or 48 h. Our results revealed significantly reduced BMDM migration following infection with all *Leishmania* spp. Evaluated compared to uninfected controls at both 24 (Figure 2B) and 48 h (Figure 2C). To further confirm these findings, we then evaluated human macrophages in migration assays using a transwell system with MCP-1 chemoattractant. These results again indicated a significant reduction in macrophage migration following infection with all three *Leishmania* spp. at both 24 (Figure 2D) and 48 h post-infection (Figure 2E).

### 3.3 *Leishmania* infection reduces adhesion complex formation within macrophages in a 3D environment

**FIGURE 3:**
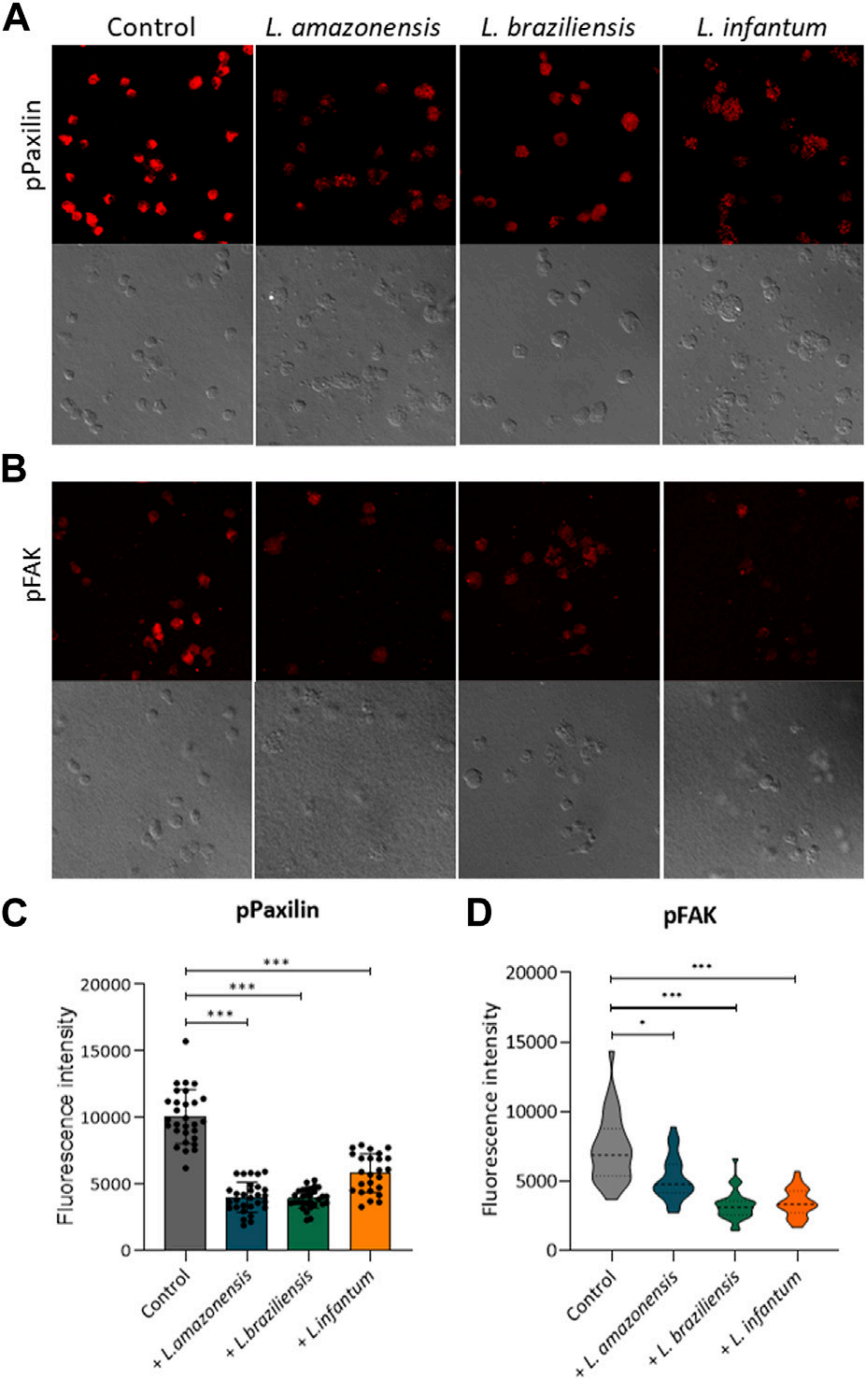
*Leishmania* infection reduces adhesion complex formation in murine macrophages. BMDM infected or not by *L. amazonensis*, *L. braziliensis* or *L. infantum* were embedded in a collagen I matrix and immunostained with anti-pFAK and anti-paxillin antibodies. **(A)** Expression of pPaxillin fluorescence by BMDM in a 3D environment. **(B)** pFAK fluorescence expression by BMDM in a 3D environment. **(C)** pPaxillin fluorescence in BMDM in a 3D environment. **(D)** Quantification of pFAK fluorescence in BMDM in a 3D environment. Fluorescence intensity quantified by averaging 25 to 30 cells per experimental group using FIJI software. ***, *p* < 0.001 (Kruskal–Wallis; ANOVA). Results are representative of three independent experiments.

**FIGURE 4:**
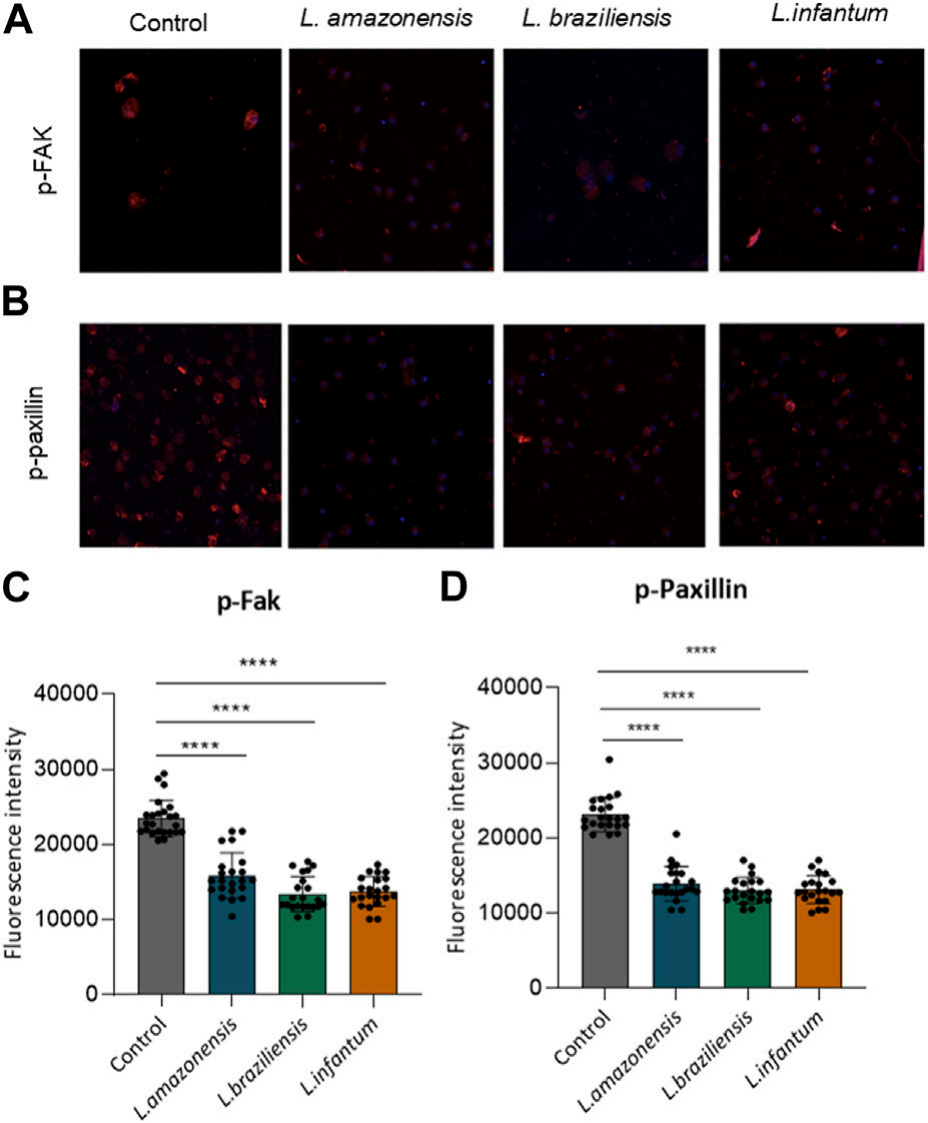
*Leishmania* infection reduces adhesion complex formation in human macrophages. Human macrophages embedded in a collagen I matrix were infected or not by *L. amazonensis*, *L. braziliensis* or *L. infantum*, then immunostained with pFAK and pPaxillin antibodies. **(A)** Expression of pFAK fluorescence in human macrophages in a 3D environment. **(B)** Fluorescence expression of pPaxillin in human macrophages in a 3D environment. **(C)** Quantification of pPaxillin fluorescence in human macrophages in a 3D environment. **(D)** Quantification of pFAK fluorescence in human macrophages in a 3D environment. Fluorescence intensity quantified by averaging 25 to 30 cells per experimental group using FIJI software. ***, *p* < 0.001 (Kruskal–Wallis; ANOVA). Results are representative of three independent experiments.

To investigate the mechanisms underlying migration, we analyzed adhesion complex formation using phosphorylated FAK and paxillin fluorescence expression in murine and human macrophages infected with *Leishmania* in a 3D environment. Our results revealed significant reductions in both phosphorylated FAK and paxillin expression in murine (Figure 3) and human macrophages (Figure 4) infected with all *Leishmania* spp. Evaluated.

### 3.4 *Leishmania* infection alters actin dynamics in macrophages in a 3D environment

**FIGURE 5:**
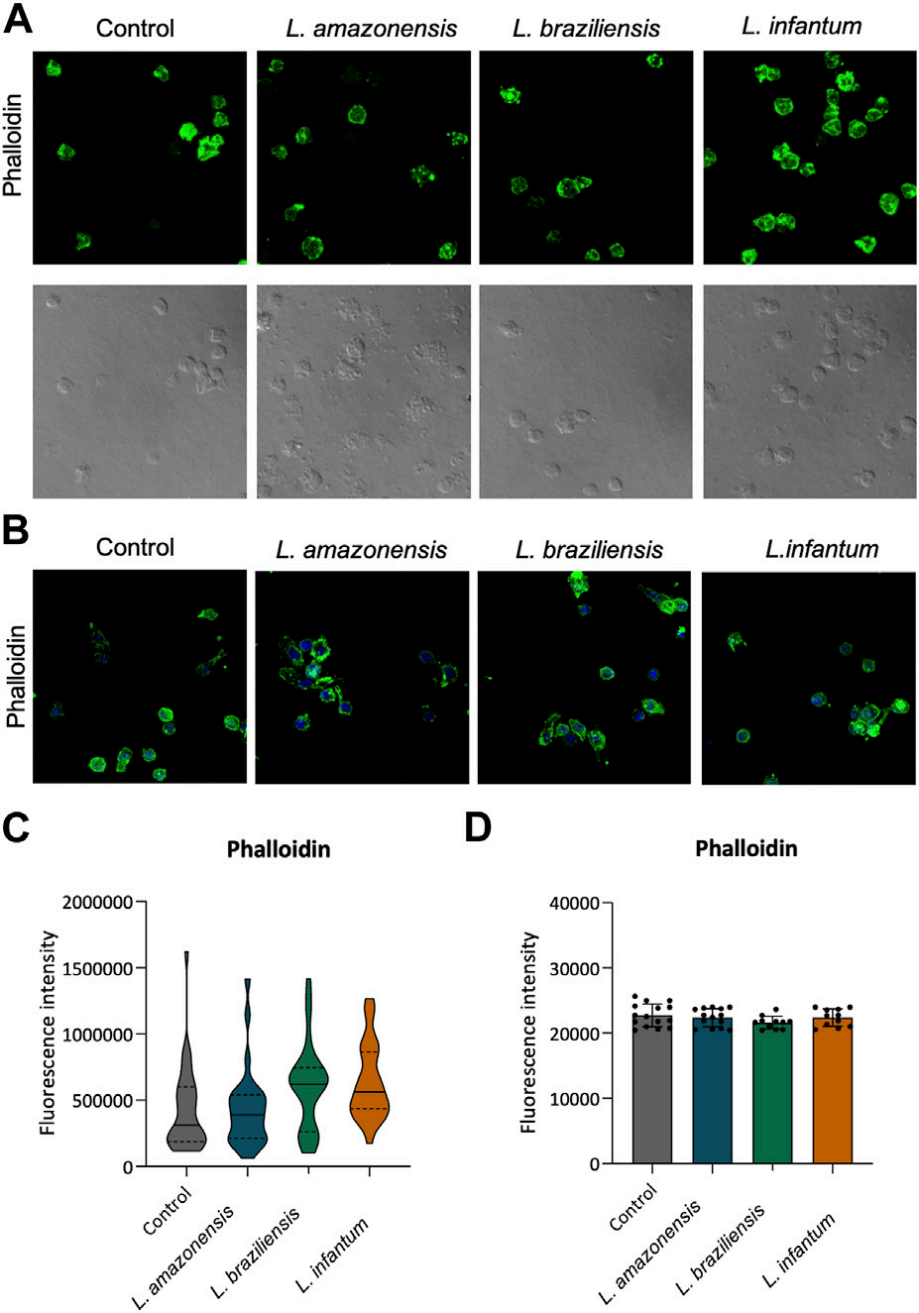
*Leishmania* spp. Infection does not alter F-actin expression in BMDM and human macrophages. Macrophages embedded in a collagen I matrix, whether infected or not by *L. amazonensis*, *L. braziliensis* or *L. infantum*, were immunostained with fluorescent phalloidin to visualize F-actin. **(A)** Expression of phalloidin fluorescence in murine macrophages in a 3D environment. **(B)** Expression of phalloidin fluorescence in human macrophages in a 3D environment. Blue: Dapi. Images were obtained using a Leica SP8 confocal microscope. **(C)** Quantification of F-actin fluorescence in murine macrophages in a 3D environment. **(D)** Quantification of F-actin fluorescence in human macrophages in a 3D environment. Fluorescence intensity quantified by averaging 25 to 30 cells per experimental group using FIJI software. ***, *p* < 0.001 (Kruskal–Wallis; ANOVA). Representative results from three independent experiments.

**FIGURE 6:**
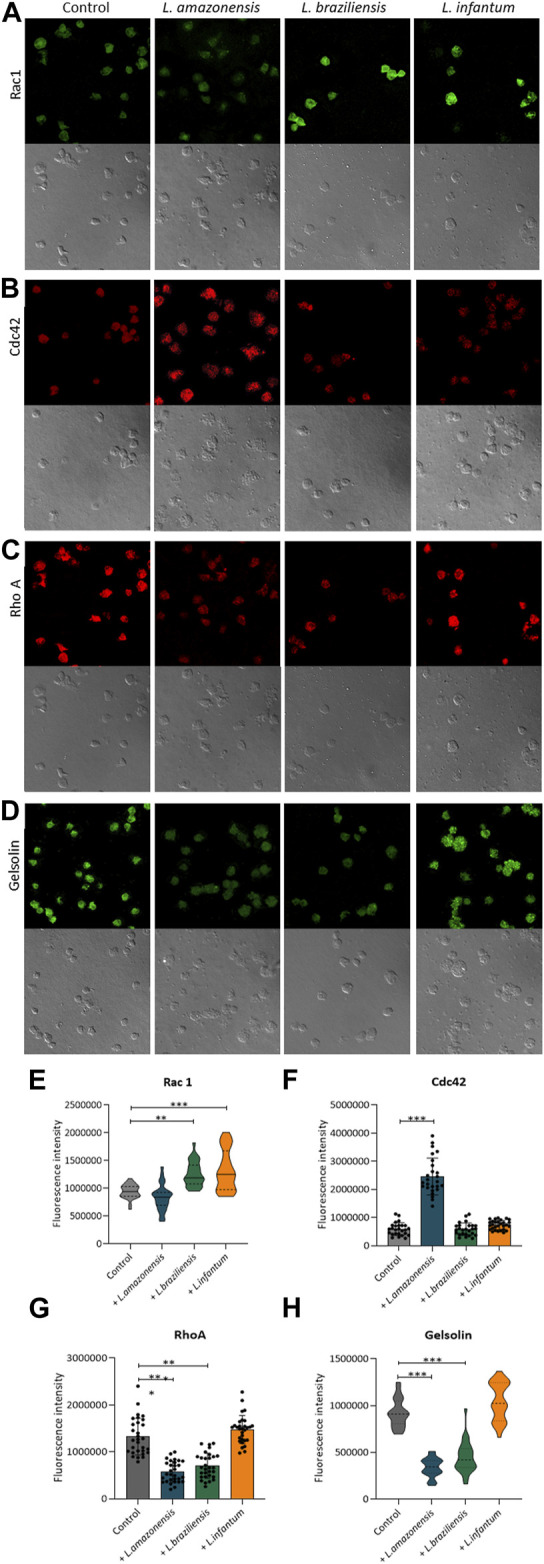
*Leishmania* infection alters actin dynamics in murine macrophages. BMDM embedded in collagen I matrix, infected or not by *L. amazonensis*, *L. braziliensis* or *L. infantum,* were immunostained with anti-Rac1, anti-Cdc42, anti-RhoA, and anti-Gelsolin. **(A)** Expression of Rac1 fluorescence in murine macrophages in a 3D environment. **(B)** Expression of Cdc42 fluorescence in human macrophages in a 3D environment. **(C)** Expression of RhoA fluorescence in murine macrophages in a 3D environment. **(D)** Expression of gelsolin fluorescence in murine macrophages in a 3D environment. Images obtained using a Leica SP8 confocal microscope. **(E)** Quantification of Rac1 **(E)**, Cdc42 **(F)**, RhoA **(G)** and Gelsolin **(H)** fluorescence in murine macrophages in a 3D environment. Fluorescence intensity quantified by averaging 25 to 30 cells per experimental group using FIJI software. ***, *p* < 0.001 (Kruskal–Wallis; ANOVA). Representative results from three independent experiments.

**FIGURE 7:**
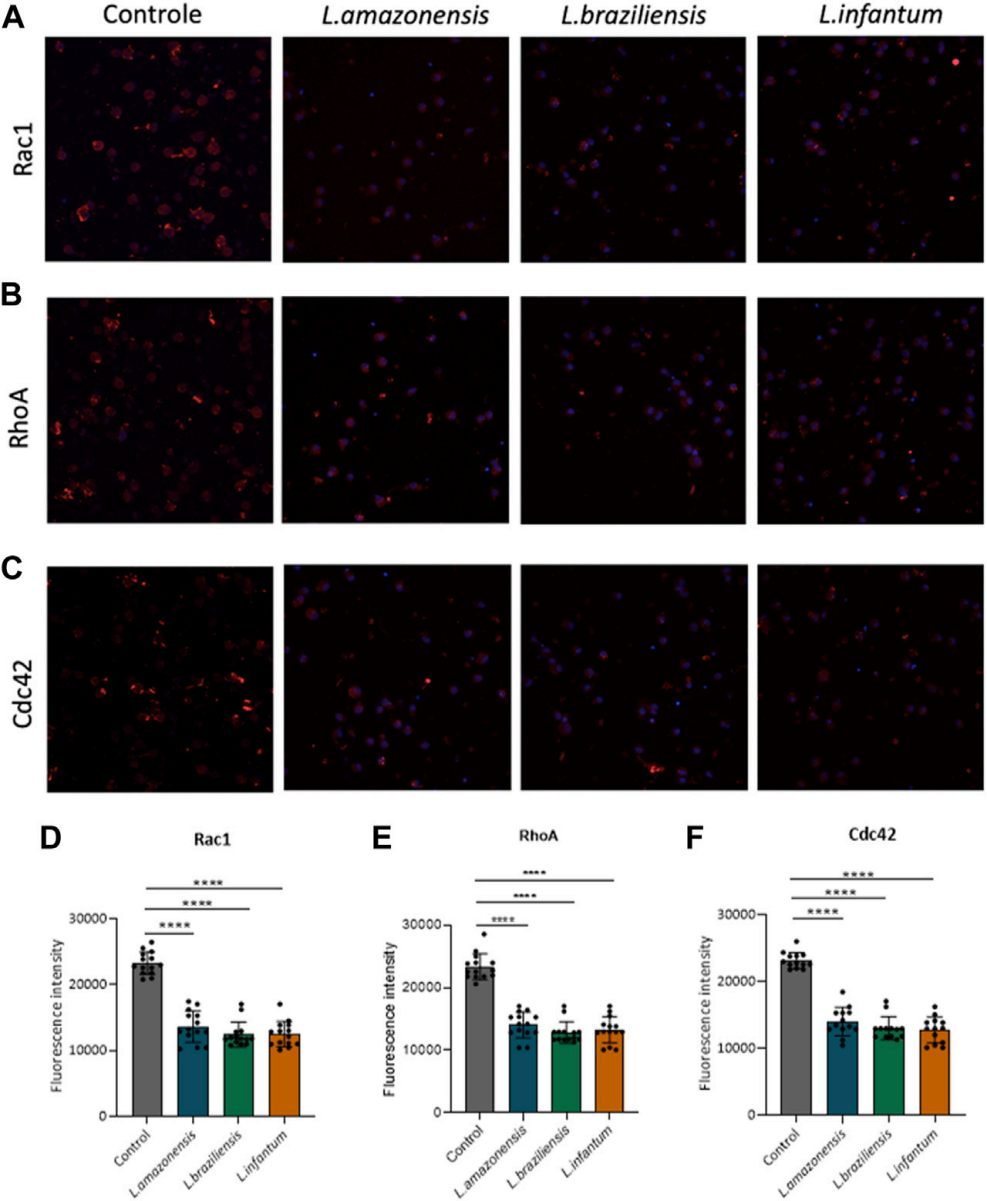
*Leishmania* infection in human macrophages reduces the expression of proteins involved in actin dynamics. Human macrophages embedded in a collagen I matrix, infected or not by *L. amazonensis*, *L. braziliensis* or *L. infantum*, were immunostained with anti-Rac1, anti-Cdc42, and anti-RhoA antibodies. **(A)** Expression of Rac1 fluorescence in human macrophages in a 3D environment. **(B)** Fluorescence expression of Cdc42 in human macrophages in a 3D environment. **(C)** Expression of RhoA fluorescence in human macrophages in a 3D environment. Images obtained using a Leica SP8 confocal microscope. **(D)** Significantly reduced Rac1 fluorescence intensity in infected human macrophages compared to controls in a 3D environment. **(E)** Significantly reduced Cdc42 fluorescence intensity in infected human macrophages compared to controls in a 3D environment. **(F)** Significantly reduced RhoA fluorescence intensity in infected human macrophages compared to controls in a 3D environment. Fluorescence intensity quantified by averaging 25 to 30 cells per experimental group using FIJI software. Blue: Dapi. ***, *p* < 0.001 (Kruskal–Wallis; ANOVA). Representative results from three independent experiments.

To investigate the role of actin cytoskeleton dynamics with respect to macrophage migration, we evaluated F-actin expression, as well as the expression of Rac1, Cdc42, RhoA, and gelsolin, in a 3D environment. Our findings indicated no differences in F-actin expression in BMDM (Figures 5A,C) or human macrophages (Figures 5B,D) infected with any of the three *Leishmania* spp. Evaluated. By contrast, murine macrophages exhibited increased Rac-1 expression after infection with *L. braziliensis* and *L. infantum* (Figures 6A,E), while *L. amazonensis* infection was found to increase Cdc42 expression (Figures 6B,F). Additionally, infection with *L. amazonensis* or *L. braziliensis* led to reduced RhoA and gelsolin expression in BMDM (Figures 6C,D,G,H). Similarly, in human macrophages, infection with *L. amazonensis*, *L. braziliensis* or *L. infantum* resulted in reduced Rac1, Cdc42, and RhoA expression in a 3D environment compared to uninfected controls (Figure 7).

### 3.5 Rates of dendritic cell infection over time, comparing *L. amazonensis*, *L. braziliensis* and *L. infantum*

**FIGURE 8:**
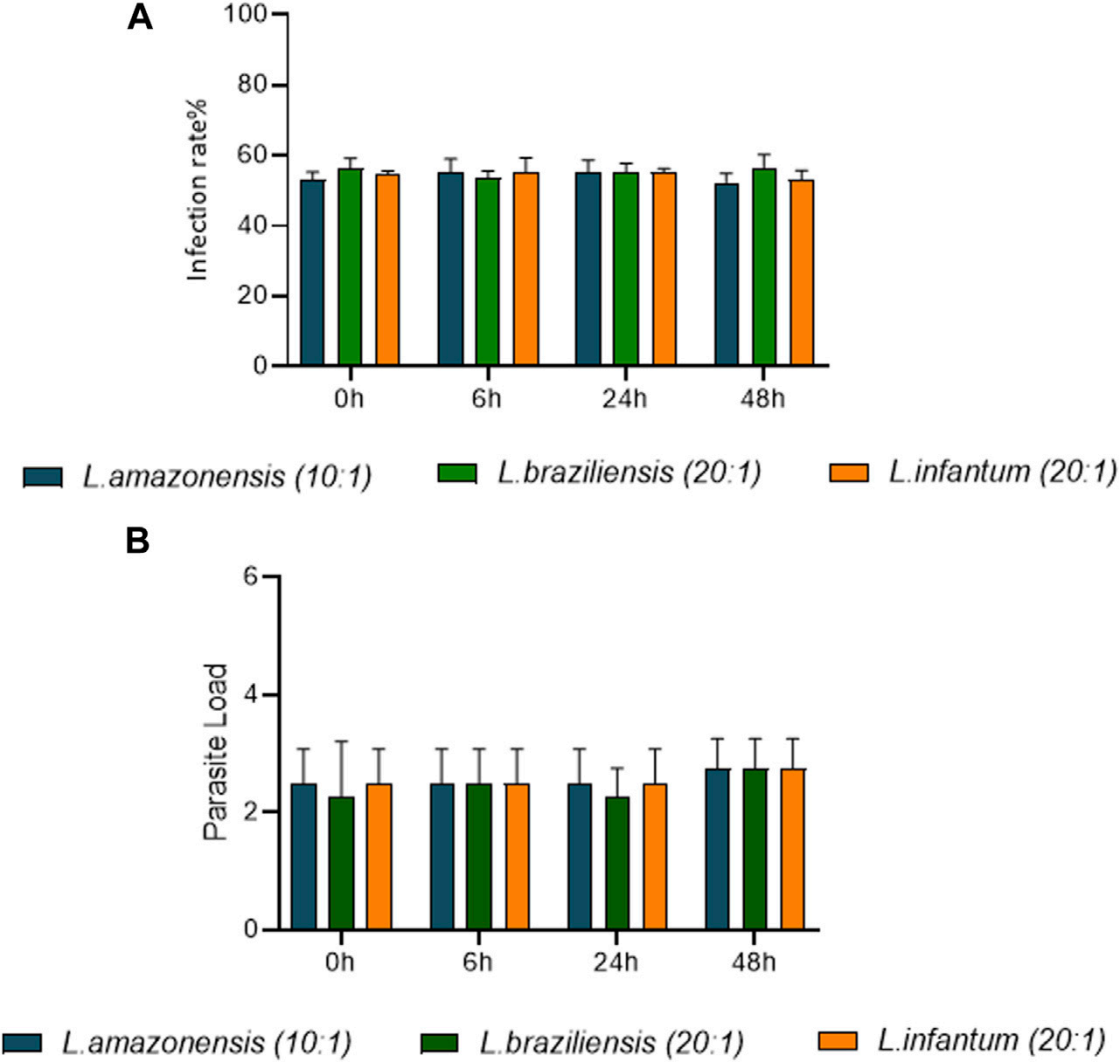
Rates of human dendritic cell infection over time, comparing *L. amazonensis, L. braziliensis and L. infantum*. Human dendritic cells infected by *L. amazonensis* (10:1), *L. braziliensis* (10:1), or *L. infantum* (20:1), 400 cells were randomly evaluated by fluorescence microscopy using DAPI. **(A)** Percentage of human dendritic cells infected by *L. amazonensis, L. braziliensis*, or *L. infantum* at 0, 6, 24 and 48 h. **(B)** Mean number of *L. amazonensis, L. braziliensis,* or *L. infantum* amastigotes per dendritic cell at 0, 6, 24 and 48 h (ANOVA). Representative results from three independent experiments.

Human dendritic cell infection rates were compared following infection by *L. amazonensis* (10:1), *L. braziliensis* (10:1) or *L. infantum* (20:1). Our results indicate that similar rates of infection (60%) were achieved between human dendritic cells infected by *L. amazonensis* (10:1), *L. braziliensis* (10:1) or *L. infantum* (20:1) at 0 h (Figure 8A). Furthermore, the number of *Leishmania* per cell was similar, on average 2 parasites per cell (Figure 8B).

### 3.6 *Leishmania infantum* induces dendritic cell migration in a 3D environment

**FIGURE 9:**
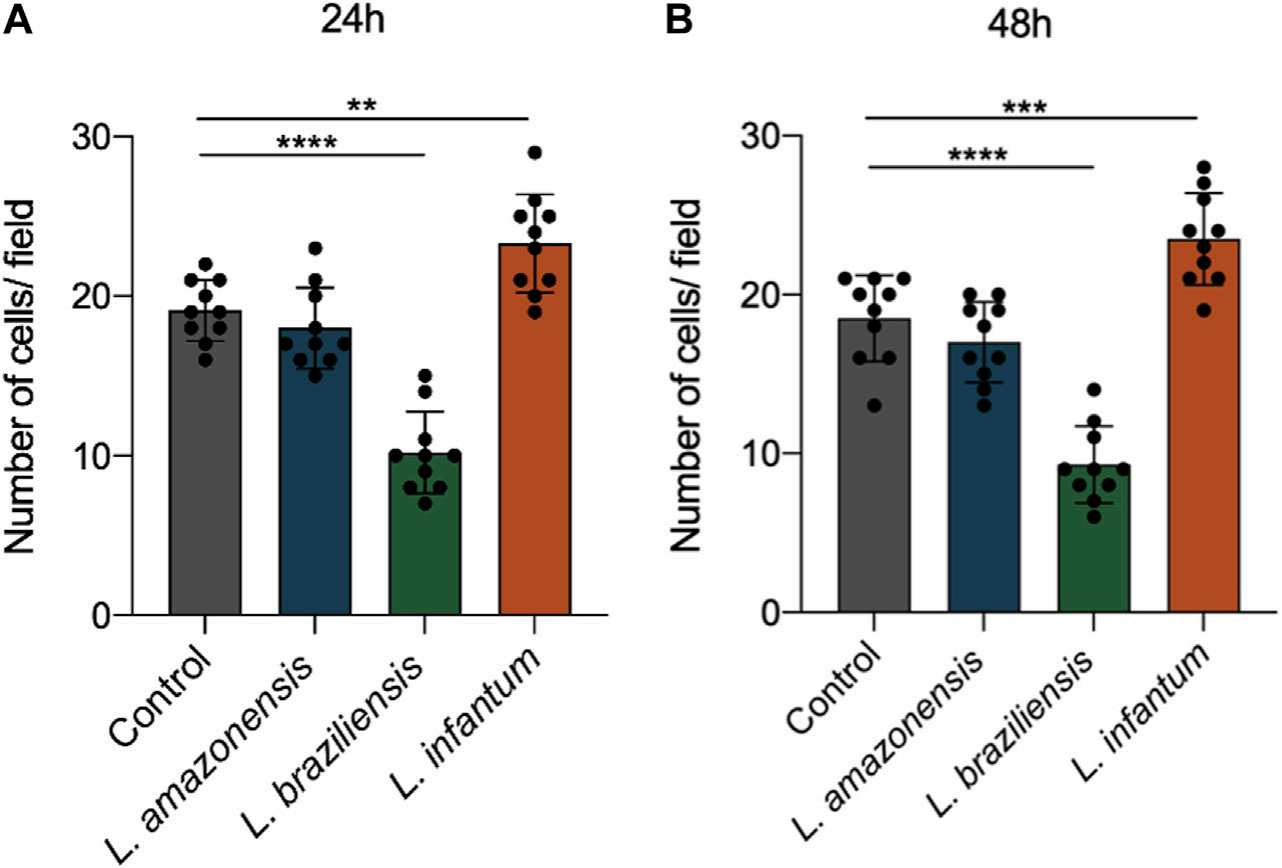
*L. infantum* increases human dendritic cell 3D migration in a 3D environment. Cells (infected or not) were incorporated to a collagen matrix. At 24 and 48 h after infection, the cells were placed in a Boyden chamber and allowed to migrate towards CCL3. Migrated cells were randomly counted with nuclear staining with DAPI through fluorescence microscopy. **(A)** Number of human dendritic cells that migrated after 24 h. **(B)** Number of human dendritic cells that migrated after 48 h ****p < 0000.1 (Student’s t-test). Representative results from three independent experiments.

To assess dendritic cell migration in a 3D environment, these cells were incorporated into a collagen I matrix and allowed to migrate for 24 or 48 h in migration assays using a transwell system. Our results revealed significantly enhanced dendritic cell migration following infection with *L. infantum* compared to *L. amazonensis* and *L. braziliensis*-infected cells, as well as uninfected controls, at both 24 (Figure 9A) and 48 h (Figure 9B). Additionally, infection with *L. braziliensis* led to reduced dendritic cell migration at both 24 (Figure 9A) and 48 h (Figure 9B).

### 3.7 *Leishmania infantum* induces adhesion complex formation within dendritic cells in a 3D environment

**FIGURE 10:**
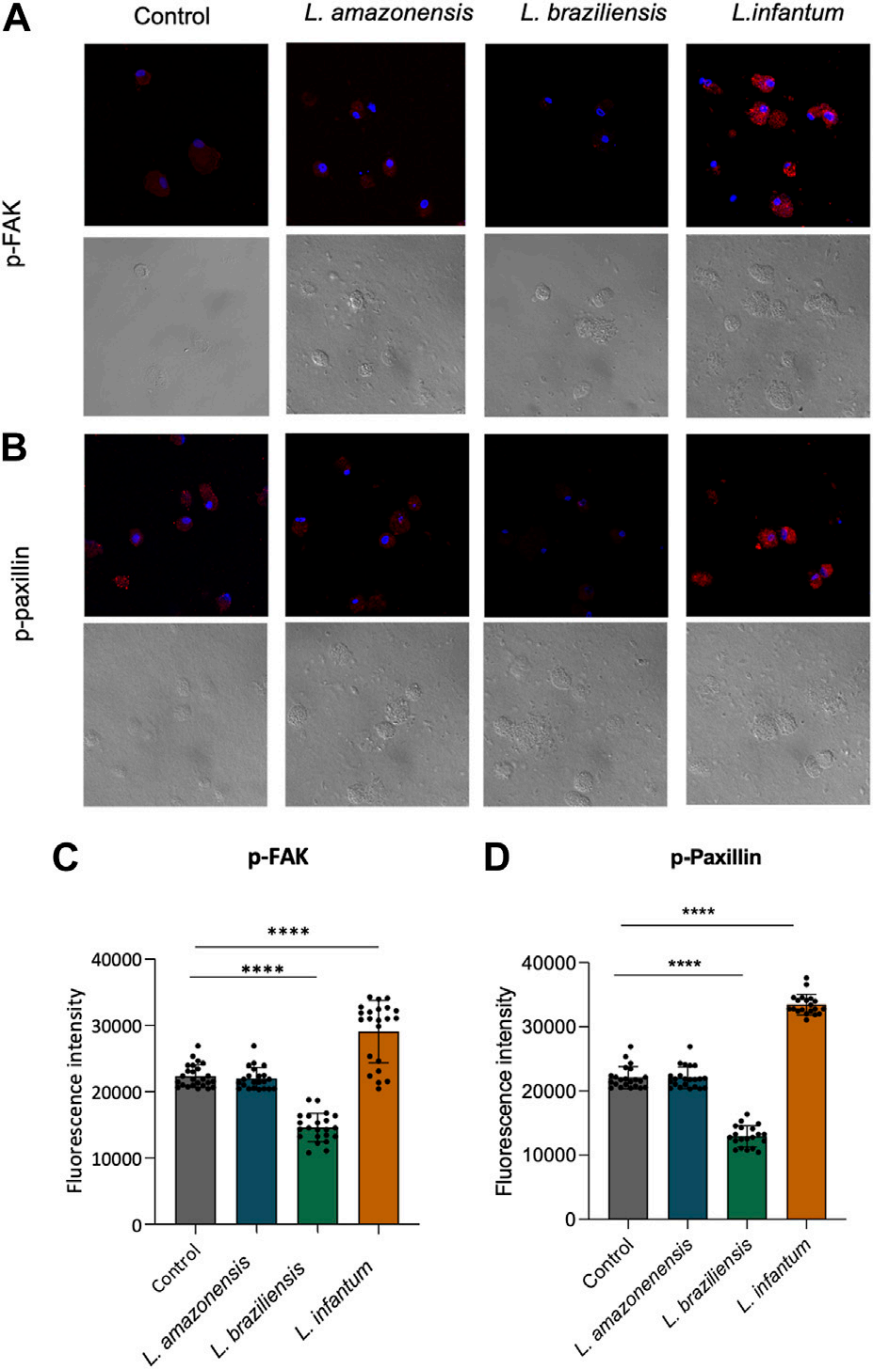
*L. infantum* induces human dendritic cell adhesion complexes formation in a 3D environment. Human dendritic cells infected or not by *L. amazonensis*, *L. braziliensis*, or *L. infantum* and embedded in a collagen I matrix were immunostained with anti-pFAK and anti-paxillin antibodies. **(A)** Expression of pFAK fluorescence in human dendritic cells in a 3D environment. **(B)** Fluorescence expression of pPaxillin in human dendritic cells in a 3D environment. **(C)** Quantification of pFAK fluorescence in human dendritic cells in a 3D environment. **(D)** Quantification of pPaxillin fluorescence in human dendritic cells in a 3D environment. Fluorescence intensity quantified by averaging 25 to 30 cells per experimental group using FIJI software. Red: anti-FAK or anti-paxillin; Blue: Dapi. ****p < 000.1 (Student’s t-test). Representative results from three independent experiments.

To investigate the mechanisms underlying dendritic cell migration, we evaluated adhesion complex formation in human dendritic cells infected with *Leishmania infantum* in a 3D environment by analyzing the expression of FAK and paxillin. Our fluorescence data analysis revealed significant increases in both FAK and paxillin in *L. infantum-*infected dendritic cells compared to uninfected controls. Additionally, a significant decrease in the expression of these proteins was noted in dendritic cells infected by either *L. braziliensis* or *L. amazonensis* compared to controls (Figures 10A–D).

### 3.8 *Leishmania infantum* alters actin dynamics in dendritic cells in a 3D environment

**FIGURE 11:**
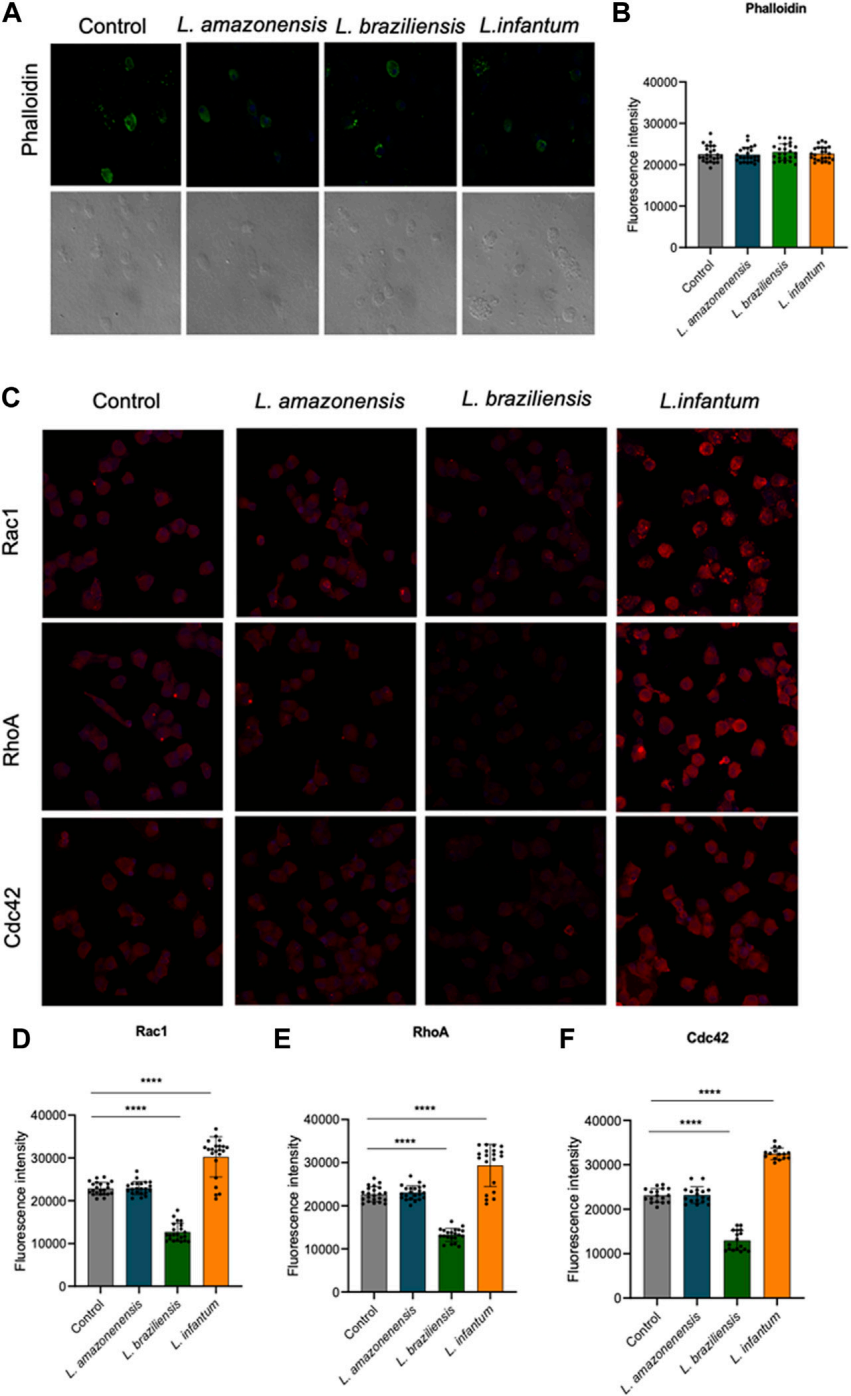
*L. infantum* increases the expression of proteins involved in human dendritic cells 3D actin polymerization. Human dendritic embedded in a collagen I matrix, whether infected or not by *L. amazonensis*, *L. braziliensis* or *L. infantum*, were immunostained with fluorescent phalloidin to visualize F-actin and anti-Rac1, anti-Cdc42 and anti-RhoA. **(A)** Expression of phalloidin fluorescence in human dendritic cells in a 3D environment. **(B)** Quantification of F-actin fluorescence in human dendritic cells in a 3D environment. **(C)** Expression of Rac1, RhoA and Cdc42 fluorescence in human dendritic cells in a 3D environment. Quantification of Rac1 **(D)**, RhoA **(E)** and Cdc42 **(F)** fluorescence in human dendritic cells in a 3D environment. Fluorescence intensity quantified by averaging 25 to 30 cells per experimental group using FIJI software. Green:phalloidin, Red: anti-Rac1, anti-RhoA, anti-Cdc42; Blue:Dapi. ***p < 0.001 (Student’s t-test). Representative results from three independent experiments.

We assessed the expression of F-actin, Rac1, Cdc42, RhoA, and gelsolin in a 3D environment to study the participation of actin cytoskeleton dynamics in dendritic cell migration. Our results showed no differences in F-actin expression between human dendritic cells infected with either of the three *Leishmania* spp. Evaluated (Figures 11A,B). However, infection with *L. infantum,* but not with *L. amazonensis* or *L. braziliensis*, led to increased Rac1, Cdc42, and RhoA expression compared to uninfected controls in a 3D environment (Figures 11C–F).

## 4 Discussion

The dissemination of *Leishmania* within the host subsequent to infection can result in a diverse range of clinical manifestations, varying from spontaneously healing skin ulcers to widespread lesions or disfiguring mucosal lesions; in extreme cases, visceral leishmaniasis can be lethal. The present study demonstrated that infection by three different species of *Leishmania* resulted in reduced macrophage migration in a 3D environment. In contrast, our results demonstrated that *L. infantum* infection resulted in increased dendritic cell migration, while *L. braziliensis* infection led to reduced dendritic cell migration. However, infection with *L. amazonensis* did not significantly affect cell migration compared to uninfected controls. Our findings, which show that *L. infantum* infection can potentially enhance the migratory capacity of dendritic cells from the site of infection, are consistent with the visceral form of the disease associated with this parasite species. Moreover, the observed changes were associated with marked differences in adhesion complex formation and actin dynamics in infected macrophages and dendritic cells.

Previous studies have shown that *Leishmania* infection inhibits the macrophage’s ability to adhere to different substrates, such as collagen, laminin, and fibronectin (Carvalhal et al., 2004; Pinheiro et al., 2006). It has also been demonstrated that infection with *L. amazonensis* leads to a reduction in macrophage migration in a two-dimensional environment (Blewett et al., 1971; Bray et al., 1983; de Menezes et al., 2017). In contrast, studies using *L. infantum* have shown an increase in BMDM migration following infection, via a PI3K-dependent pathway (Rocha et al., 2020). Additionally, studies have demonstrated that, following *Leishmania* infection, dendritic cells are able to migrate to draining lymph nodes, enabling antigen transportation (León et al., 2007; Lai et al., 2008). Furthermore, a recent work published by our group showed that *L. infantum*, but not *L. amazonensis* or *L. braziliensis*, the latter two being localized forms of CL, induces dendritic cell migration in a two-dimensional environment (Rebouças et al., 2021). Alterations in the migration of infected dendritic cells may significantly impact host immune response to *Leishmania*. This was highlighted by a recent study reporting that *L. amazonensis* infection inhibited the migration of dendritic cells from the site of infection to draining lymph nodes (Hermida et al., 2014). Although numerous reports have investigated leukocyte migration in two-dimensional environments, the study of migration using a 3D matrix has been relatively limited. Given that 3D migration occurs within tissues, *in vitro* models that mimic three-dimensional extracellular environments can provide valuable insight into phagocyte migration. To our knowledge, this is the first study attempting to examine the migration of both *Leishmania*-infected macrophages and dendritic cells in an three-dimensional environment *in vitro* (Ley et al., 2007; Vérollet et al., 2011).

Our results evidenced reduced 3D migration in both murine and human *Leishmania*-infected macrophages, in association with lower FAK and paxillin expression, which stands in agreement with the data obtained from experimentation in a 2D environment by de Menezes et al. (2017). We also identified increased 3D migration of human dendritic cells associated with the expression of the same adhesion complex proteins, which is consistent with data previously published by our group in a 2D environment (Rebouças et al., 2021). The phosphorylation of FAK plays a fundamental role in cell adhesion, since its activation leads to the phosphorylation and the consequent activation of paxillin, a key protein in cellular adhesion and migration (Schaller and Parsons, 1995). Studies using embryonic mouse fibroblasts mutated for FAK, to which paxillin cannot bind, have demonstrated the loss of localization and phosphorylation of FAK in focal adhesion, resulting in altered migration dynamics and inhibited cell adhesion (Deramaudt et al., 2014). In a study involving *T. cruzi*, it was observed that this pathogen is capable of increasing FAK activation, thus increasing the incidence of cardiomyopathy as well as suggesting an increase in disease dissemination (Melo et al., 2019).

The actin cytoskeleton is crucial for cell migration, with the Rho GTPase family of proteins playing a key role in the regulation of this dynamic process (Ridley and Hall, 1992). The inhibition of Rho GTPases was shown to provoke deficiencies in cell migration (Sander et al., 1999). Herein we observed increased Rac1 expression in BMDMs infected with *L. braziliensis* and *L. infantum*; by contrast, infection by *L. amazonensis* resulted in increased Cdc42 expression. It is possible that the enhanced expression of these GTPases in macrophages may be associated with increased actin dynamics at the leading edge of murine macrophages. However, we also identified reduced expression of Rac1 and Cdc42 following infection by all three *Leishmania* spp. In human macrophages, which suggests a reduction in actin dynamics at the leading edge of these cells. The alterations in actin dynamics observed in murine macrophages infected by *Leishmania* in a three-dimensional environment corroborate previously published data from experiments conducted in a two-dimensional environment by de Menezes et al. (2017). Additionally, it has been demonstrated that Cdc42 and Rac1 promote actin polymerization through the Arp2/3 complex by activating their effector proteins, N-WASP and WAVE-2, respectively (Spiering and Hodgson, 2011). Inhibition of Cdc42 has also been associated with reduced recruitment and chemotaxis of macrophages during invasion and metastasis (Zhang et al., 2021). Furthermore, a previous study has reported that macrophages deficient in Rac1 lose their ability to invade dense matrix environments such as Matrigel (Wheeler et al., 2006). Moreover, the differences observed in GTPases between murine and human macrophages could be related to the fact that murine macrophages were stimulated with MCSF during their differentiation (Pixley, 2012). Further studies are required to investigate the state of activation of these GTPases in both cell types. In addition, the present results further identified increased Rac1 and Cdc42 expression in human dendritic cells infected with *L. infantum*, but not *L. amazonensis* or *L. braziliensis*, which suggests increased F-actin turnover in these cells. This finding is consistent with our previous results conducted in a two-dimensional environment (Rebouças et al., 2021), leading us to speculate that *L. infantum* may facilitate the migration of infected dendritic cells to the draining lymph nodes. Of note, previously published work was shown to induce dendritic cell migration upon *Toxoplasma gondii* infection, thereby potentiating parasite dissemination (Lambert et al., 2006). In addition, RhoA, a member of the Rho GTPase family, plays a crucial role in regulating cell migration by mediating the formation of stress fibers in the cell body and posterior region (Ridley and Hall, 1992). Previous studies have also shown that RhoA activity is associated with reduced cell protrusion (Nobes and Hall, 1999; Rottner et al., 1999). Additionally, ROCK activation by RhoA can inhibit actin polymerization (Nobes and Hall, 1999). Furthermore, decreased levels of active GTP-bound RhoA in macrophages, caused by increased cholesterol in the cell membrane, hinder cell migration (Nagao et al., 2007). Our findings indicate reduced RhoA expression in murine macrophages infected with *L. amazonensis* and *L. braziliensis*, as well as in human macrophages infected with all three *Leishmania* spp. Evaluated. This finding provides evidence of the potential suppression of RhoA activity by these *Leishmania* species, which may result in decreased formation of stress fibers and cell protusion, potentially impairing cell migration. In contrast, we also demonstrated that infection with *L. infantum* led to increased RhoA expression in human dendritic cells, which could favor cell migration. Our study further investigated the expression of gelsolin, an actin-binding protein known to facilitate F-actin breakage (McGough et al., 2003; Silacci et al., 2004; Khaitlina et al., 2013). Previous studies have reported that *Yersinia*’s YopO protein can phosphorylate gelsolin, leading to actin binding and separation, which ultimately results in the alteration of actin dynamics in phagocytic cells (Singaravelu et al., 2017). As our study demonstrated a reduction in gelsolin expression in murine macrophages infected with *L. amazonensis* and *L. braziliensis*, this could lead to decreased F-actin breakage in these cells, which may also be responsible for the reduced migration capacity observed in BMDM.

Taken together, the present study showed that *Leishmania* spp. Infection leads to decreased cell migration in both murine and human macrophages, indicating the critical role of these cells in controlling parasites at the lesion site. The present findings also provide evidence of increased human dendritic cell migration following *L. infantum* infection, suggesting a potential association between this cell type and disease visceralization. Elucidating the mechanisms underlying the migration of *Leishmania*-infected cells and their potential role in lesion development and parasite dissemination is crucial to understanding the pathogenesis of leishmaniasis. The findings presented herein serve to expand the base of knowledge on host cell migration during the course of *Leishmania* infection, as well as the consequent dissemination of the parasites within the vertebrate host.

## Data Availability

The original contributions presented in the study are included in the article/Supplementary Material, further inquiries can be directed to the corresponding author.
