# Supplementary figures and images for: Leishmania infection alters macrophage and dendritic cell migration in a three-dimensional environment
Source: Front Cell Dev Biol. 2023 Jul 28;11:1206049. doi: 10.3389/fcell.2023.1206049 (PMC10416637; doi:10.3389/fcell.2023.1206049)

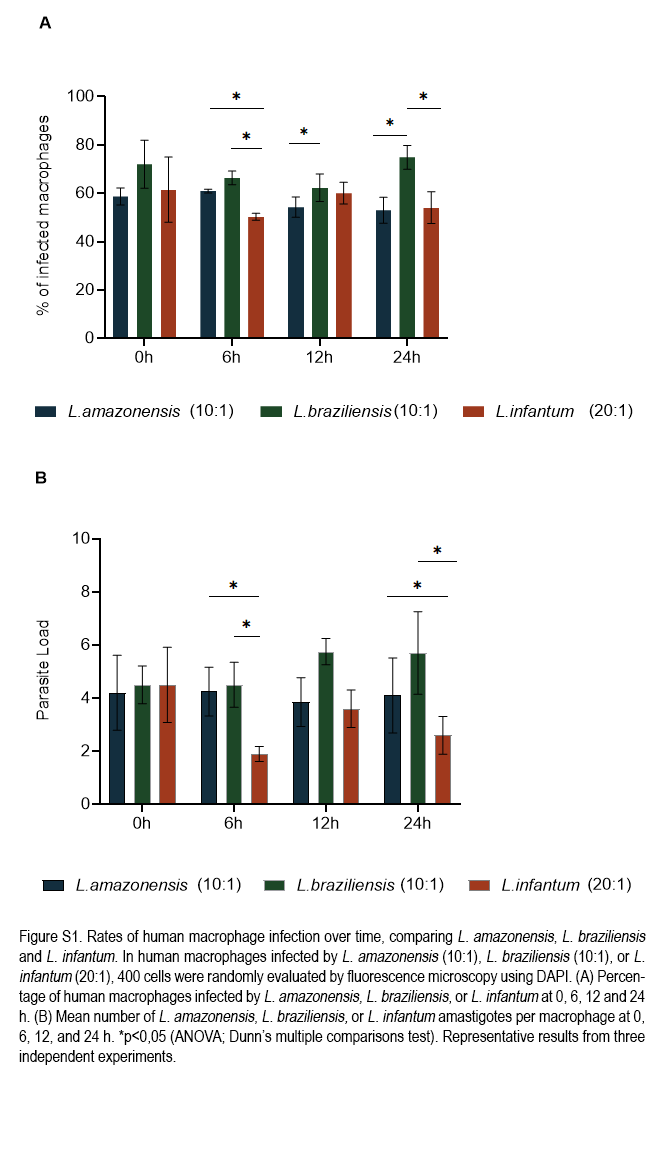

Supplement: Supplementary file 1 [file Image1.TIFF]
